# Does pre-diagnostic loss to follow-up among presumptive TB patients differ by type of health facility? An operational research from Hwange, Zimbabwe in 2017

**DOI:** 10.11604/pamj.2018.31.196.15848

**Published:** 2018-11-21

**Authors:** Munekayi Padingani, Ajay Kumar, Jaya Prasad Tripathy, Nyasha Masuka, Sidingiliswe Khumalo

**Affiliations:** 1 Ministry of Health and Child Care Zimbabwe, Provincial Medical Directorate, Matebeleland North Province, Harare, Zimbabwe; 2 International Union Against Tuberculosis and Lung Disease, Paris, France; 3 International Union Against Tuberculosis and Lung Disease, South-East Asia Office, New Delhi, India

**Keywords:** Pre-diagnostic loss to follow up, pre-treatment loss to follow-up, initial default, public-private-for-profit mix, attrition, SORT IT, operational research

## Abstract

**Introduction:**

While there are many studies assessing the pre-treatment loss to follow-up (LFU) among tuberculosis patients in public sector, there is no evidence from private-for-profit health sector and pre-diagnostic LFU from Zimbabwe. We aimed to assess the gaps in the cascade of care of presumptive TB patients registered during January-June 2017 in different types of health facilities in Hwange district, Zimbabwe.

**Methods:**

This was a cohort study involving review of routine programme data. Pre-diagnostic LFU was defined as the proportion of presumptive TB patients not tested using sputum microscopy or Xpert MTB/RIF. A log binomial regression was done to assess factors associated with pre-diagnostic LFU.

**Results:**

Of 1279 presumptive TB patients, 955(75%) were tested for TB and 102(8%) were diagnosed as having TB. All TB patients were started on treatment. Pre-diagnostic LFU (overall 25%) was significantly higher among patients visiting private-for-profit health facilities (36%), local self-government run council health facilities (35%) and church-run mission health facilities (25%) compared to government health facilities (14%). Pre-diagnostic LFU was significantly higher among patients in rural areas (30%) compared to urban areas (18%). Type of health facility was associated with pre-diagnostic LFU after adjusting for HIV status and area of residence.

**Conclusion:**

While pre-diagnostic LFU was high, there was no pre-treatment LFU. Pre-diagnostic LFU was especially high in private-for-profit and council health facilities and rural areas. National TB Programme should take immediate steps to improve access in rural areas and support the private-for-profit and council health facilities by improving sputum collection and transport.

## Introduction

Tuberculosis (TB) remains a major global health problem affecting millions of people each year and is one of the top ten causes of deaths worldwide. TB is now the leading cause of death from a single infectious agent, ranking above HIV/AIDS [1]. In 2016, of the 10.4 million estimated new patients globally, only 6.3 million were detected and officially notified, leaving a gap of 4.1 million patients [1]. This means that an alarming 40% of the patients were not visible to the public health system. Unless we are able to identify these 'missing' millions and place them on appropriate treatment, we will not be able to reach the targets envisioned by the World Health Organization's 'End TB strategy' and United Nation's Sustainable Development Goals (SDGs) [2, 3]. Zimbabwe is one of the 30 high TB burden countries [1]. In terms of missing TB patients, of the estimated 34,000 new TB patients in 2016, only 27,353 (80%) were notified leaving behind a gap of about 20% [1]. There might be many possible reasons for this gap which include i) Difficulties in accessing health facilities ii) Pre-diagnostic loss to follow-up (TB patients reaching the health facilities, but not investigated and diagnosed) iii) Pre-treatment loss to follow-up (TB patients diagnosed, but not initiated on treatment) and iv) Non-notification of TB patients diagnosed and treated in the public-private-mix facilities.

Several studies conducted across the globe have shown that the pre-treatment loss to follow-up(LFU) among TB patients varied from 4% to 38% and was more common in settings from Africa (18%, 95% CI: 13-22) compared to Asia (13%, 95% CI: 10-15) [4]. All these studies are from the public health sector and there is very limited evidence from the private-for-profit health sector on this issue. A study from Pakistan in 2017 by Khan *et al* showed that an alarming 64% of smear-positive TB patients diagnosed in public-private-for-profit facilities of Lahore city in Pakistan were lost to follow-up before treatment [5]. While there are many studies on pre-treatment LFU, the evidence on pre-diagnostic LFU is scarce. Two studies from South Africa reported that about 18% of the presumptive TB patients were not tested for TB and estimated that as a result, about 5% of TB patients did not get diagnosed even after reaching the health facility [6, 7]. There is limited evidence on this issue from Zimbabwe. A study conducted by Chadambuka *et al* in 2005 showed that 27% of smear-positive TB patients were not started on treatment [8]. There is no evidence on pre-diagnostic LFU and there has not been any study from the private-for-profit health sector in Zimbabwe. Thus, we conducted this operational research to assess the gaps in the cascade of care of presumptive TB patients in the public and private-for-profit health facilities in a selected district of Zimbabwe. The gaps thus identified will help plan targeted interventions to reduce attrition along the cascade of TB care. The specific objectives were: among patients with presumptive TB attending the different types of private-for-profit health facilities of Hwange District, Zimbabwe between January and June 2017, to determine the i) number and proportion tested for TB and factors associated with non-testing, ii) number and proportion diagnosed as TB (bacteriologically confirmed and clinical TB) and factors associated with it, iii) number and proportion initiated on treatment among those diagnosed above, and iv) time interval between identification of presumptive TB and testing and between date of diagnosis and TB treatment initiation.

## Methods

**Study design:** This was a cohort study involving analysis of secondary data collected routinely by the national TB programme.

### Setting

**General setting:** Zimbabwe is a landlocked country in southern Africa known for its dramatic landscape and diverse wildlife. The country is divided into 10 provinces and 59 districts with a population of 13,061,239 [9]. With a gross domestic product of 16.29 billion USD, Zimbabwe is considered one of the low income countries [10]. The country has a poor life expectancy of 61 years largely driven by deaths due to infectious diseases such as tuberculosis, HIV/AIDS, influenza and pneumonia [9]. Access to quality health care is a major challenge in areas outside the two major cities of Harare and Bulawayo, especially the southern part of country where populations are scattered with some communities needing to travel more than 70 kilometres to access primary health care services.

**Specific setting:** The study was conducted in Hwange district of Matebeleland North Province, which is one of the country's eight rural provinces, located in the North West part of the country and has a population of about 800,000 [8]. The province has seven districts out of which Hwange is the study district. Hwange is predominantly a rural district with a population of about 141,000 [8]. It is served by 45 health facilities. Among the health facilities, 17 are private-for-profit, 16 are government health facilities (run by the Ministry of Health), seven are council health facilities (run by the local self-governments), three are mission health facilities (run by church organisations) and two serve the uniform forces. The district has six TB diagnostic centres (including two private-for-profit health facilities) where sputum smear microscopy is performed and among the six, two perform Xpert MTB/RIF assay as well. One of the Xpert machines is situated in a private-for-profit health facility.

**Zimbabwe NTP:** The country has a National TB Control Programme (NTP) with officers at the central level in Harare, provincial levels including metropolitan cities, district levels, primary care levels and community level with diagnostic and treatment services integrated into general health care [11]. TB case finding is passive and diagnosis is established through sputum smear microscopy, Xpert MTB/RIF and chest radiography for pulmonary tuberculosis and other investigations for extra-pulmonary disease (based on the site affected). All presumptive TB patients (defined as anybody with cough, fever, weight loss, night sweats) identified are registered in presumptive TB register [11]. This register is available at all health facilities including private-for-profit health facilities. The sputum specimens are collected by laboratory staff or clinicians from the identified presumptive TB patients and either tested in the same facility (if available) or transported by an environmental health technician (EHT present in government health facilities) to the nearest designated laboratory for sputum smear microscopy and/or Xpert MTB/RIF. For other health facilities, a designated person from the government travels around the district to all health facilities on a motorcycle, collects the sputum specimens and transports them to the designated diagnostic facility. Sputum collection and transportation is done once a week. The results of the tests are documented in the laboratory register and shared back to the referring health facility. The results including the laboratory number are then documented in the presumptive TB register by the TB nurse. Those who are diagnosed with TB by sputum microscopy and/or Xpert MTB/RIF are initiated on treatment using standardized regimens as per national and international guidelines and are registered in the TB register which is maintained by the TB nurse at clinic level and by the District TB Coordinator at District level and are monitored for treatment outcomes. Patients who are bacteriologically negative, but with chest radiography findings suggestive of TB are diagnosed as "clinical TB" and started on treatment. TB treatment is available at all health facilities [11]. TB care (including laboratory tools and consumables, drugs, records and registers) in the private-for-profit health facilities is provided and monitored by the NTP. In Zimbabwe, by law, TB treatment is provided only by the NTP. All investigations and treatment are provided free of charge to the patients, irrespective of the health facility accessed.

**Study population:** Presumptive TB patients registered in all the health facilities of Hwange district, Zimbabwe from January to June 2017 were included.

**Data variables, sources of data and data collection:** Data were collected using a structured proforma by visiting all the health facilities between September 2017 and January 2018. At each health facility, we referred to the presumptive TB register to fill up the data collection proforma. The laboratory registers were cross-checked to complete the list. If a patient was found in the laboratory register but not in the presumptive register, then he/she was included in the study population. Then we looked at the laboratory register of the designated laboratory to assess if the patient was tested or not. The tracking of patients across registers was done using name, age, sex and laboratory number (wherever available) and within a period of eight weeks after the date of identification of presumptive TB patient. For patients diagnosed as bacteriologically confirmed TB, TB register was reviewed to assess if treatment had been initiated or not. We again used the patient's name, age and sex as tracking variables and tracked for a period of eight weeks after date of diagnosis in the TB register. We also reviewed the TB register to identify any clinically diagnosed TB patients from the cohort of presumptive TB patients included in the study and started on treatment. We considered a perfect match if there was a match of laboratory number or name and sex or name and age (plus or minus three years). We took the assistance of the district TB coordinator and the TB nurse at each health facility to navigate the records and extract the study variables.

**Analysis and statistics:** Data were single-entered from the structured proforma and analysed using Epi Data software (version 3.1 for entry and v 2.2.2183 for analysis, Epi Data Association, Odense, Denmark). There were two key outcomes in the study: 1) Pre-diagnostic LFU: proportion of presumptive TB patients not tested for TB by either sputum microscopy or Xpert MTB/RIF assay within eight weeks of identification. 2) Pre-treatment LFU: Proportion of bacteriologically confirmed patients not started on TB treatment within eight weeks of diagnosis. The duration between presumptive TB identification and testing and diagnosis and treatment start was summarized using median and interquartile range. A multivariable regression analysis (log binomial regression) was conducted to explore the factors associated with pre-diagnostic LFU and TB diagnosis and presented as relative risks (RRs) and 95% confidence intervals (CIs). A p value of <0.05 was considered statistically significant.

**Ethics:** Permission to access programme records was obtained from the Provincial Medical Director (Matebeleland North Province), National Tuberculosis Control Programme Zimbabwe. Ethics approval was obtained from Medical Research Council of Zimbabwe and The Union Ethics Advisory Group, Paris, France.

## Results

Of all the health facilities, there were no presumptive TB patients documented in the seven private-for-profit and two uniform forces health facilities. In the remaining 36 health facilities, there were 1279 presumptive TB patients (57% female and median age of 38 years). Of them, 6% were from private-for-profit health facilities, 40% each were from government and council health facilities and 15% from mission health facilities. Majority of the patients were from rural (59%) areas, although, the patients attending private-for-profit health facilities were mostly urban (94%) (Table 1).

**Table 1:** Demographic characteristics of presumptive TB patients, stratified by the type of health facility, in Hwange district, Zimbabwe, 2017

Variable	Private-for-profit health facilities	Government health facilities	Council health facilities	Mission health facilities	Total
	N (%)	N (%)	N (%)	N (%)	N (%)
Number of presumptive TB patients	83 (100)	509(100)	491(100)	196(100)	1279(100)
Gender*					
Female	51(61)	304(60)	268(55)	103(53)	726(57)
Male	32(39)	203(40)	223(45)	93(47)	551(43)
Not recorded	0(0)	2(<1)	0(0)	0(0)	2(<1)
Age*					
0-14 years	1(1)	43(8)	29(6)	8(4)	81(6)
15-44 years	57(69)	294(58)	289(59)	109(56)	749(59)
45 and above	25(30)	172(34)	173(35)	79(40)	449(35)
Residence*					
Rural	4(5)	310(61)	315(64)	123(63)	752(59)
Urban	78(94)	191(37)	174(35)	73(37)	516(40)
Not recorded	1(1)	8(2)	2(1)	0(0)	11(1)

TB=Tuberculosis

**Figure 1:**
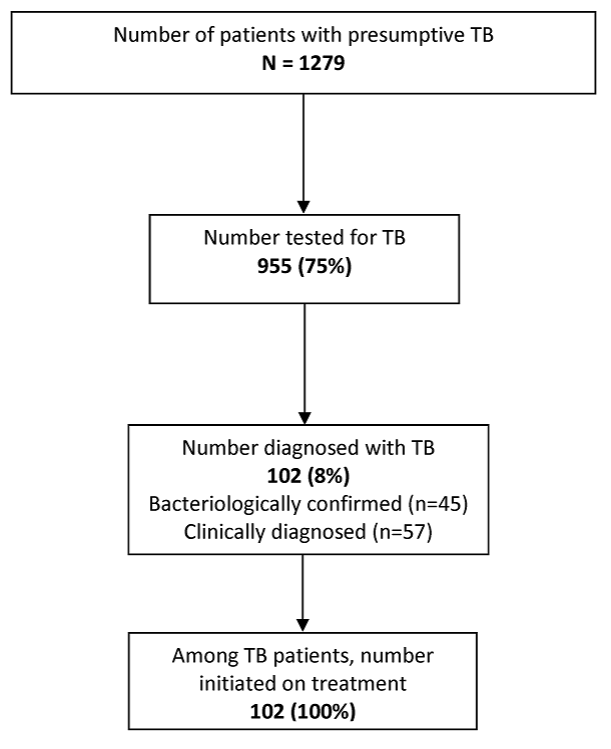
TB diagnostic and treatment cascade among presumptive TB patients registered health facilities of Hwange district, Zimbabwe, 2017

**TB diagnosis and treatment cascade:** Of the 1279 presumptive TB patients, 955 (75%) were tested for tuberculosis using either sputum microscopy (n=474) or Xpert MTB/RIF (n=477) or both (n=4). Thus, the pre-diagnostic LFU was 25%. A total of 102(8%) TB patients were diagnosed-14 sputum positive TB, 31 Xpert positive (which includes one with rifampicin resistance) and 57 clinically diagnosed TB. All 102 TB patients were initiated on TB treatment (Figure 1). About half (52%) of all study participants were HIV positive and this varied between private-for-profit (36%) and other health facilities (~54%). Table 2 shows the variations in the extent of testing, diagnosis and treatment among the various types of health facilities. The proportions tested for TB and diagnosed with TB respectively were highest among patients attending government health facilities (86%, 10%) and the mission health facilities (75%, 11%). Private-for-profit health facilities had the lowest testing and diagnosis rates (64%, 4%) followed closely by the council hospitals (65%, 5%).

**Table 2:** Clinical characteristics of presumptive TB patients, stratified by the type of health facility, in Hwange district, Zimbabwe, 2017

Variable	Private-for-profit health facilities	Government health facilities	Council health facilities	Mission health facilities	Total
	N(%)	N(%)	N(%)	N(%)	N(%)
Number of presumptive TB patients	83(100)	509(100)	491(100)	196(100)	1279(100)
HIV status (%)*					
Negative	33(40)	149(29)	178(36)	71(36)	431(34)
Positive	30(36)	294(58)	256(52)	93(47)	673(52)
Not recorded	20(24)	66(13)	57(12)	32(17)	175(14)
Tested for TB using sputum microscopy or Xpert MTB/RIF (%)*					
Tested	53(64)	436(86)	320(65)	146(75)	955(75)
Not tested	30(36)	73(14)	171(35)	50(25)	324(25)
TB status (%)*					
TB	3(4)	52(10)	26(5)	21(11)	102(8)
No TB	80(96)	457(90)	465(95)	175(89)	1177(92)
Time to diagnosis (days)@					
Median (IQR)	2 (0-4)	1 (0-3)	2 (1-5)	1 (0-4)	1 (0-4)
Number of TB patients	3	52	26	21	102
TB type (%)*#					
Bacteriologically Confirmed	67	39	50	48	44
Clinically confirmed	33	61	50	52	56
TB treatment (%)*#	100	100	100	100	100
**Time to treatment (days)#$**					
Median (IQR)	0(0-0)	0 (0-2)	0 (0-2)	1(0-1)	0 (0-2)

* Column percentages

# For these indicators, the denominator is the total number of TB patients

@ data on dates of presumptive TB registration and date of testing available for 907 presumptive TB patients only

$ data on dates of diagnosis and treatment available for 78 TB patients only

TB=Tuberculosis; IQR=Interquartile range; HIV=Human Immunodeficiency Virus

**Time to diagnosis and treatment:** the overall median (Inter Quartile Range) time to diagnosis from being registered as presumptive TB patient was 1(0-2) days and time to treatment from diagnosis was 0 (0-2) days and did not vary significantly by the type of health facility (Table 2).

**Factors associated with pre-diagnostic LFU:** Adjusted analysis showed that patient's residence, HIV status and type of health facility were independently associated with pre-diagnostic LFU (Table 3). Participants residing in rural areas were twice more likely to be LFU than those from urban areas. HIV positive presumptive tuberculosis patients were 40% more likely to be LFU than HIV negative patients. Patients attending private-for-profit health facilities were nearly four times more likely to be LFU than those attending government health facilities.

**Table 3:** Demographic and clinical factors associated with pre-diagnostic loss to follow-up (LFU) among presumptive TB patients in Hwange district, Zimbabwe, 2017

Characteristics	Total N	Pre-diagnostic LFU n(%)	RR (95% CI)	aRR (95% CI)	
Total	1279	324(25)				
**Gender**					
Female	726	191	(26)	Ref	Not included
Male	551	133	(24)	0.9 (0.7-1.1)	
**Age group**					
0-14 years	81	24	(30)	Ref	Not included
15-44 years	749	192	(26)	0.9 (0.6-1.2)	
45 years and above	449	108	(24)	0.8 (0.6-1.2)	
**Residence**					
Urban	516	94	(18)	Ref	Ref
Rural	752	228	(30)	1.7 (1.4-2.0)	2.1 (1.6-2.6)
Not recorded	11	2	(18)	1.0 (0.3-3.5)	1.4 (0.4-4.6)
**HIV status**					
Negative	431	95	(22)	Ref	Ref
Positive	673	173	(26)	1.2 (0.9-1.5)	1.4 (1.1-1.7)
Not recorded	175	56	(32)	1.5 (1.1-1.9)	1.7 (1.3-2.2)
**Type of health facility**					
Government	509	73	(14)	Ref	Ref
Private-for-profit	83	30	(36)	2.5 (1.8-3.6)	3.7 (2.5-5.3)
Council	491	171	(35)	2.4 (1.9-3.1)	2.5 (2.0-3.2)
Mission	196	50	(26)	1.8 (1.3-2.5)	1.6 (1.2-2.2)

HIV=Human Immunodeficiency Virus; TB=Tuberculosis; RR=Unadjusted Relative Risk; aRR=Adjusted Relative Risk; CI=Confidence Interval; Ref= Reference category RR in bold font indicate statistically significant results (p<0.05)

**Factors associated with TB diagnosis:** Presumptive TB patients from urban areas who were tested for TB were three times more likely to have TB compared to rural areas. Those attending private-for-profit and council health facilities were significantly less likely to have TB compared to government health facilities Table 4.

**Table 4:** Demographic and clinical factors associated with TB diagnosis among presumptive TB patients in Hwange district, Zimbabwe, 2017

Characteristics	Total N	TB Diagnosis N (%)		RR (95% CI)	aRR (95% CI)
Total	1279	102	(100)		
**Gender**					
Female	726	49	(7)	Ref	Ref
Male	551	52	(9)	1.4 (1.0-2.0)	1.3 (0.9-1.8)
**Age group**					
0-14 years	81	5	(6)	Ref	Not included
15-44 years	749	79	(10)	1.7 (0.7-4.1)	
45 years and above	449	18	(4)	0.7 (0.3-1.7)	
**Residence**					
Rural	752	33	(4)	Ref	Ref
Urban	516	67	(13)	3.0 (2.0-4.4)	3.1 (2.0-4.6)
Not recorded	11	2	(18)	4.1 (1.1-15.2)	2.7 (0.6-12.6)
**HIV Status**					
Negative	431	24	(6)	Ref	Ref
Positive	673	67	(10)	1.8 (1.1-2.8)	1.4 (0.9-2.3)
Not recorded	175	11	(6)	1.1 (0.6-2.3)	0.9 (0.4-1.7)
**Type of health facility**					
Government	509	52	(10)	Ref	Ref
Private-for-profit	83	3	(4)	0.4 (0.1-1.1)	0.2 (0.1-0.8)
council	491	26	(5)	0.5 (0.3-0.9)	0.5 (0.3-0.8)
Mission	196	21	(11)	1.1 (0.7-1.7)	1.0 (0.7-1.7)

HIV=Human Immunodeficiency Virus; TB=Tuberculosis; RR=Unadjusted Relative Risk; aRR=Adjusted Relative Risk; CI=Confidence Interval; Ref= Reference category

RR in bold font indicate statistically significant results (p<0.05)

## Discussion

This is the first study from Zimbabwe looking at the cascade of TB diagnosis and treatment among presumptive TB patients, stratified by type of health facilities. Our study revealed that one-quarter of the presumptive TB patients was lost before testing. Significant pre-diagnostic LFU of around 18% was also reported from previous studies in South Africa [6]. Pre-diagnostic loss to follow up (LFU) was more in private-for-profit and council health facilities where more than one-thirds of patients were lost before testing. This might be because, most patients from the private-for-profit health institutions after being registered as a presumptive TB patient are referred to the government facilities for testing as they do not have sputum collection and transport facility. We speculate that most of these referred patients may not reach the government health facility for testing and are thus lost. EHTs are involved in TB diagnosis and treatment in the government health facilities. Absence of those staff in private-for-profit and some council health institutions might also be a reason for poor follow-up of presumptive TB patients. High pre-diagnostic LFU in rural areas could be attributed to poor access to TB diagnostic facilities as there is only two TB diagnostic facility in rural areas. The study also reported poor bacteriological positivity among those tested for TB. We hypothesize the following four reasons for this finding: i) poor specimen quality as the specimens are transported once a week to the nearest diagnostic facility raising concern regarding proper storage and transportation of sputum samples, ii) limited availability of Xpert testing services and hence all HIV-infected presumptive TB patients did not undergo Xpert testing. In some of the HIV-infected patients who underwent sputum smear microscopy, we might have missed diagnosing TB [12] iii) HIV patients with possible active TB might have died before getting tested, and iv) inability on the part of the providers (especially those in the private-for-profit and council health facilities) to correctly identify presumptive TB patients. All patients diagnosed with TB were initiated on TB treatment which is quite encouraging compared to other studies in this region. In contrast to this finding, a systematic review by MacPherson *et al*. has reported high pre-treatment LFU of 18% in the African region [4]. A study in Zimbabwe by Chadambuka *et al*. reported that 27% of smear-positive TB patients were not started on treatment [8]. Some of the possible reasons for no pre-treatment LFU might be: i) low numbers of TB patients in the district allowing staff to prioritize attention to TB care, ii) dedicated staff (TB nurse) present in most health facilities to monitor TB treatment (initiation and completion), and iii) better supervision and monitoring with onsite data verification and urgent corrective action.

The study had some strengths. First, the study covered all health facilities in the district including the private-for-profit health facilities. Second, the study captured information from a large cohort of presumptive TB patients in the routine programme setting, thus reflecting the true scenario in the field. Third, we adhered to the Strengthening the Reporting of Observational studies in Epidemiology (STROBE) guidelines to report the study findings [13]. Fourth, multiple registers were cross-checked to collect information on all types of TB including clinical/extra-pulmonary TB thus giving a complete picture of all TB patients compared to other previous studies on this issue. The study had some limitations as well. First, this being a study conducted in a single district, we should be careful in generalizing the study findings to other settings. Second, we did not investigate the reasons for the high pre-diagnostic loss to follow-up. Third, it might be possible that some of the presumptive TB patients were not documented in the presumptive TB register. This may have led to an underestimation of pre-diagnostic LFU. However, we enrolled patients directly from the laboratory register even though they were not recorded in the presumptive register. This might have mitigated the limitation to some extent. This study has several programmatic implications. First, there is a need to improve access to TB diagnostic services in private-for-profit health facilities where there is high pre-diagnostic LFU. Sputum collection and transportation could be an answer to that which has been shown to improve access to TB testing and diagnosis [14, 15]. Being already implemented in the government facilities, could be extended to cover private-for-profit facilities. In rural areas, more facilities should be equipped with TB diagnostic services to provide better access. Currently, there are only two health facilities which provide TB diagnosis (sputum microscopy) to 66,525 people in the rural areas. Second, the sputum collection and transport should be available as and when required rather than once a week in order to improve the quality of specimens for better yield. Further research is warranted to document the quality of specimen tested through the sputum collection and transport mechanism. Patients should be instructed and supervised by the providers to produce good quality, mucoid sputum specimen. Third, qualitative research needs to be undertaken to explore the reasons for high pre-diagnostic LFU and understand the perspectives of various stakeholders.

## Conclusion

There is high pre-diagnostic LFU especially in the private-for-profit health facilities and in rural areas. But, there was no pre-treatment LFU. Access to TB diagnosis could be improved by equipping more facilities with TB diagnostics in rural areas and expanding sputum transport and collection to private-for-profit facilities.

### What is known about this topic

- Systematic reviews show that pre-treatment loss to follow-up (LFU which is the gap between diagnosis and treatment) among TB patients varies from 4% to 38% and was more common in settings from Africa (18%) compared to Asia (13%);
- While there are studies assessing the pre-treatment LFU among patients in public sector, there is no evidence from private-for-profit health sector from Zimbabwe;
- There is no evidence on pre-diagnostic LFU (the gap between presumptive TB identification and testing for TB) from Zimbabwe.

### What this study adds

- In Hwange district of Zimbabwe, we found about 25% of presumptive TB patients were lost to follow-up before diagnosis (pre-diagnostic LFU). But, there was no pre-treatment LFU;
- Pre-diagnostic LFU was significantly higher among patients visiting private-for-profit-for-profit health facilities (36%), local self-government run council health facilities (35%) and church-run mission health facilities (25%) compared to government health facilities (14%);
- Pre-diagnostic LFU was significantly higher among patients in rural areas (30%) compared to urban areas (18%).

## Competing interests

The authors declare no competing interests.
